# Validation of a novel dark-blood delayed enhancement technique for the detection of papillary muscle scar

**DOI:** 10.1186/1532-429X-18-S1-Q6

**Published:** 2016-01-27

**Authors:** Dave Wendell, Elizabeth Jenista, Wolfgang G Rehwald, Han W Kim, Michele Parker, Enn-Ling Chen, Robert Judd, Raymond Kim

**Affiliations:** 1 grid.26009.3d0000000419367961Medicine/Cardiology DCMRC, Duke University, Durham, NC USA; 2 Siemens Healthcare, Chicago, IL USA

## Background

Papillary muscle scarring may result in mitral regurgitation and may be foci for ventricular arrhythmias. However, papillary muscle scarring may be difficult to detect with delayed enhancement MRI (DE-MRI) since there is often poor contrast between hyperenhanced tissue and immediately adjacent bright blood pool. We have developed a new, **F**low-**I**ndependent **D**ark-blood **D**e**L**ayed **E**nhancement technique (FIDDLE) that increases conspicuity of areas of myocardial scar adjacent to the blood pool. We validated FIDDLE in detecting papillary muscle scar in an animal model of myocardial infarction (MI) and demonstrate feasibility in patients with MI.

## Methods

We used a canine model of MI which results in both papillary muscle and LV wall infarction. CMR was performed 1 week to 6 months following MI at 3T (Siemens Verio). FIDDLE and standard DE-MRI were acquired in an interleaved fashion 10-20 minutes after Gadolinium administration (0.2 mmol/kg) using matched parameters (eg. slice thickness: 7 mm, in-plane resolution: 1.2 × 1.0, etc.). Following CMR, hearts were stained with triphenyltetrazolium chloride (TTC) to provide a gold standard histopathology reference. FIDDLE and DE were performed blinded to subject identity and pathology. MI patients had enzymatically confirmed MI and x-ray coronary angiography confirmed the infarct related artery (IRA). In patients, imaging and analysis were performed similar to that for canines.

## Results

**Figure 1:**
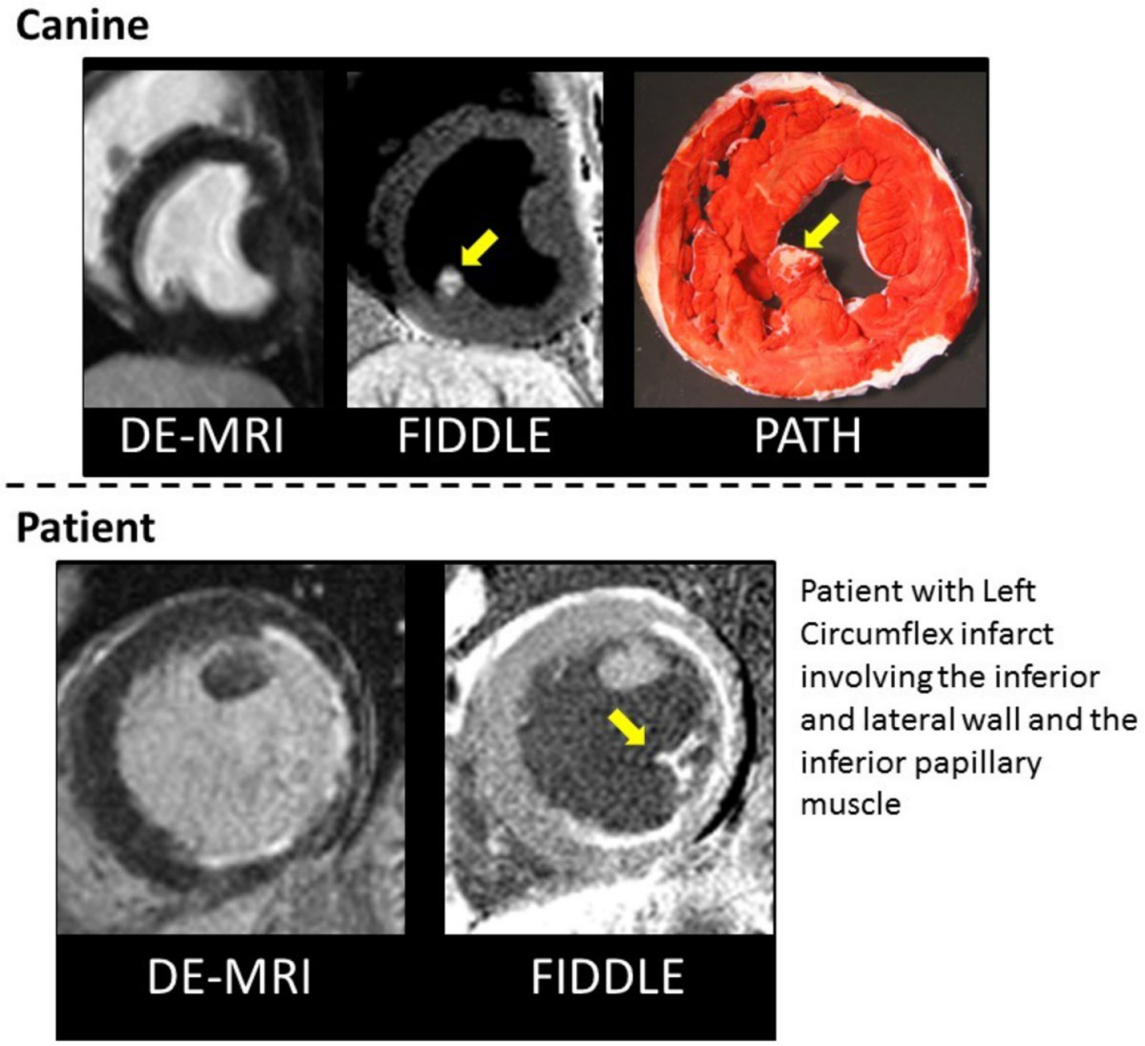
**DE-MRI (left), FIDDLE (center), and TTC (right) showing myocardial infarction of only the inferior paillary muscle (arrows)**. Bottom: patient example of left circumflex coronary artery infarction showng subendocardial hyperenhancement and papillary infarction

Black-blood images were successfully acquired with FIDDLE in all animals (n = 22) and patients (n = 19). Representative images of papillary muscle scarring are shown in Figure. FIDDLE provided improved sensitivity and accuracy compared to DE-MRI for the detection of papillary muscle scaring in animals (both p = 0.03; Table). In patients, 22/42 papillary muscles were found to have scar by FIDDLE, whereas only 2/42 were found by DE-MRI. FIDDLE identified 9 papillary muscles that were entirely scarred throughout. In these, DE-MRI not only failed to identify papillary muscle scar, but the papillary muscle itself was missed entirely (Figure 1, bottom).

## Conclusions

This is the first study comparing contrast enhanced MRI to gold standard pathology for the detection of papillary muscle scarring. We show that DE-MRI is often insensitive for detecting papillary muscle scarring. Conversely, FIDDLE is superior to DE-MRI for the detection of papillary muscle scarring, and appears to have high accuracy when compared to pathology.

**Table 1:** Animal Results

Animals	Sensitifity	Specificity	Accuracy
FIDDLE	80% (16/20)	96% (23/24)	89% (39/44)
DE-MRI	55% (11/20)	96% (23/24)	77% (34/44)
p-value	0.03	1.0	0.03
